# Identification of genetic loci for flag-leaf-related traits in wheat (*Triticum aestivum* L.) and their effects on grain yield

**DOI:** 10.3389/fpls.2022.990287

**Published:** 2022-09-08

**Authors:** Ying Wang, Ling Qiao, Chenkang Yang, Xiaohua Li, Jiajia Zhao, Bangbang Wu, Xingwei Zheng, Pengbo Li, Jun Zheng

**Affiliations:** ^1^Institute of Wheat Research, Shanxi Agricultural University, Linfen, China; ^2^School of Life Sciences, Shanxi University, Taiyuan, China; ^3^Institute of Cotton Research, Shanxi Agricultural University, Yuncheng, China

**Keywords:** wheat, flag leaf, flag leaf volume measurement, quantitative trait loci, grain

## Abstract

Flag-leaf-related traits including length (FLL), width (FLW), area (FLA), thickness (FLT), and volume (FLV) of flag leaves are the most important determinants of plant architecture and yield in wheat. Understanding the genetic basis of these traits could accelerate the breeding of high yield wheat varieties. In this study, we constructed a doubled haploid (DH) population and analyzed flag-leaf-related traits in five experimental locations/years using the wheat 90K single-nucleotide polymorphism array. It’s worth noting that a novel method was used to measure FLT and FLV easily. Leaf thickness at two-thirds of the leaf length from tip to collar represented the average leaf thickness as measured with freehand sections and was used to calculate the leaf volume. In addition, flag-leaf-related traits showed positive correlations with yield related traits under two different water regimes. A total of 79 quantitative trait loci (QTL) controlling the five traits were detected among all chromosomes except 4D and 5A, explaining 3.09–14.52% of the phenotypic variation. Among them, 15 stable QTL were identified in more than three environments, including two major QTL for FLT, six for FLW, three for FLA, two for FLT and two for FLV. DH lines with positive alleles at both QTL regions had an average FLL (9.90%), FLW (32.87%), FLT (6.62%), FLA (18.47%), and FLV (20.87%) greater than lines with contrasting alleles. *QFLT-2B*, *QFLV-2A*, and *QFLV-7D* were co-located with yield-related traits. The 15 QTL were validated by tightly linked kompetitive allele specific PCR (KASP) markers in a recombinant inbred line (RIL) population derived from a different cross. *QFLL-4A*, *QFLW-4B*, *QFLA-5D.1*, *QFLA-7A*, *QFLA-7D.1*, *QFLT-2B*, *QFLT-6A*, *QFLV-2A*, and *QFLV-7D* are likely novel loci. These results provide a better understanding of the genetic basis underlying flag-leaf-related traits. Also, target regions for fine mapping and marker-assisted selection were identified and these will be valuable for breeding high yielding bread wheat.

## Introduction

Wheat (*Triticum aestivum* L.) is one of the most important food crops worldwide. Wheat production must be increased sustainably to meet the food demand of a growing human population. The flag leaf is the main organ for photosynthesis. It is regarded as the “functional leaf” in wheat production and contributes 45–58% of plant photosynthate during grain-filling (Duncan, 1971; Xu and Zhao, 1995; Sharma et al., 2003; Khaliq et al., 2008). Also, flag-leaf-related traits are important determinants of plant architecture and grain yield. Donald (1968) regarded upright leaves as an “ideotype” for wheat. Having vertical leaves improves sunlight capture, thus enhancing photosynthesis and the production of dry matter. Moreover, flag-leaf-related traits showed a significant correlation with traits associated with yield and quality, such as thousand grain weight (TGW), grain number per spike (GNS), grain weight per spike (GWS), grain hardness, and grain yield (Guitman et al., 1991; Sakamoto et al., 2006). Therefore, to better breed for high yield it is necessary to understand the genetic basis of flag-leaf-related traits.

Flag-leaf-related traits, including flag leaf length (FLL), flag leaf width (FLW), flag leaf area (FLA), flag leaf thickness (FLT), and flag leaf volume (FLV), are complex quantitative traits controlled by multiple genes and are strongly influenced by environmental factors (Simón, 1999; Coleman et al., 2001; Kobayashi et al., 2003). Many quantitative trait loci (QTL) for flag-leaf-related traits have been reported in wheat. A QTL for FLW on chromosome 5A and 5B was fine mapped in previous studies (Xue et al., 2013; Zhao et al., 2022). A total of 38 QTL for flag-leaf-related traits was identified in a recombinant inbred line (RIL) population, among which were three stable QTL on chromosomes 4B, 5B, and 6B (Fan et al., 2015). Another study detected 61 QTL for flag-leaf-related traits in a RIL population using a genetic linkage map that integrated high-density simple sequence repeat (SSR) and single-nucleotide polymorphism (SNP) markers (Wu et al., 2016). Twenty-three putative QTL for flag-leaf-related traits were detected in another study, among which 15 were detected in at least two environments (Liu et al., 2018). A total of 43 QTL for traits related to flag leaves and yield were identified using four RIL populations (Hu et al., 2020). Using the wheat 90K SNP array and both genome-wide association study (GWAS) analysis and bi-parental linkage mapping, 23 QTL regulating flag-leaf-related traits were detected (Yan et al., 2020). Eight stable QTL in six populations were reported by using a 55K SNP array and SSR markers (Ma et al., 2020) and eight major QTL were identified in 11 environments (Tu et al., 2021). Although QTL for FLL, FLW, and FLA have been found in previous studies, the genetic basis of FLT and FLV in common wheat has not been studied in detail.

The amount of light absorbed by the leaves and the diffusion of CO_2_ through the leaf tissue depends in part on leaf thickness (Givnish, 1979; Agustí et al., 1994; Syvertsen et al., 1995). Some studies have shown that leaf thickness is related to photosynthesis and growth rate (Poorter, 1990; Enriquez et al., 1996; Nielsen et al., 1996; Garnier et al., 1999). The number of chloroplasts can be increased by increasing leaf thickness, thus improving photosynthetic capacity (Araus et al., 1986; Devika et al., 2018). Thicker leaves can increase leaf water content under dry conditions (Wang et al., 2011). Increasing leaf mass area, leaf thickness, and stomatal closure were used to reduce water loss and achieve higher yield under drought and high temperature stress (Cellier et al., 2000; Peña-Rojas et al., 2005). In addition, leaf thickness is closely related to the vertical shape of leaves. When plant density is high, canopy structure can be changed to reduce shade and improve the photosynthetic efficiency of lower leaves (Pendleton et al., 1968; Stewart et al., 2003). QTL for leaf thickness have been reported in rice. The major QTL *qSLW-7* for flag leaf specific leaf weight was located on chromosome 7, which was very close to a major QTL for flag leaf dry weight and flag specific leaf area (Kanbe et al., 2008). A major QTL for flag specific leaf area in rice was identified and overlapped with a major QTL for chlorophyll content (Takai et al., 2010). FLT and FLV are important determinants of yield in cereal crops, yet there are few studies of these traits in wheat.

Given the importance of flag-leaf-related traits to wheat yield, it is essential to identify and pyramid major and stably expressed loci for flag-leaf-related traits from diverse wheat germplasm resources. In the present study, a simple yet accurate detection method for FLT was developed and used to calculate FLV. Using the new method and conventional methods, QTL for five flag-leaf-related traits were detected in a DH population derived from a cross between the common wheat cultivars Jinchun 7 and Jinmai 919. Results provide a basis for selecting ideal flag leaves to increase yield by improving wheat plant architecture.

## Materials and methods

### Plant materials and field trials

Two populations with Jinmai 919 as a common parent were used, comprised of 180 DH lines from a cross of Jinchun 7 × Jinmai 919 and 165 F_10_ RILs from cross of DH118 × Jinmai 919. Bred by the Institute of Maize Research, Shanxi Academy of Agricultural Sciences, Jinchun 7 is a high-yielding variety for irrigated conditions. Jinmai 919 has strong drought resistance and was developed by the Institute of Wheat Research, Shanxi Agricultural University. DH118 is also a high-yielding variety for irrigated conditions selected by the Institute of Wheat Research, Shanxi Agricultural University. Also, five wheat varieties with various plant habit and adaptation were selected for leaf thickness measurements, including Liangxing 99 (released for the National Huanghai winter wheat area), Jimai 22 (released by Shandong Province), Nongda 211 (released for the Northern Winter wheat area), Taichang 29 (a spring wheat released by Sichuan Province), and a line selected from Jinmai 919. Three flag leaves were collected for measurements 20 days after flowering from each of the five wheats.

The two populations were planted at the Yaodu Experimental Station (36°08′N, 111°52′E, altitude 450 m), the Hancun Experimental Station (36°25′N, 111°67′E, altitude 450 m), and the Yuncheng Experimental Station (34°35′ N, 110°15′ E, altitude 450 m) in Shanxi Province in 2018–2019, 2019–2020, and 2020–2021. Plants were grown under well-watered (WW) and rainfed conditions, providing five environments designated as E1 (WW, 19YD), E2 (WW, 20YD), E3 (WW 20YC), E4 (rainfed, 21HC), and E5 (rainfed, 21YD). The field design was a randomized complete block with three replications. Each plot consisted of two 1.5 m rows spaced 0.3 m apart at 21 seeds per row. Field management used standard wheat production practices.

### Testing flag-leaf-related traits

FLL, FLW, FLT, FLA, and FLV were measured on the main tiller of 10 randomly selected plants. FLL (cm) was measured from leaf collar to the tip. FLW (cm) was measured at the widest part of the leaf. The derived trait FLA (cm^2^) was calculated as FLA = FLL × FLW × 0.83 after Xue et al. (2013).

To develop a convenient way to measure FLT, the flag leaves of the five wheat varieties were cut into 20 equal segments and the cross-sectional area of each cut was calculated using the image processing software Image J.^1^ To calculate the thickness of the flag leaf the formula h = S/W was used where h is the flag leaf thickness, S is the cross-sectional area, and W leaf is leaf width at the cut. The thickness of each of the 20 segments was then compared with their average.

FLV (mm^3^) was calculated as FLV = FLT × FLA. The accuracy of the calculated FLV was verified by measuring FLV with the drainage method. A volume of water (V_1_) was placed into beakers, and a lead weight with volume V_2_ was used to sink a flag leaf segment into the water. The volume after immersion (V_3_) was recorded. FLV was calculated as FLV = V_3_ – V_2_ – V_1_ and the measurement replicated three times for each variety. Additionally, grain-yield-related traits were measured for all lines, including spikelet number per spike (SN), GNS, spikelet length (SL), grain length (GL), grain width (GW), grain thickness (GT), TGW, and plant height (PH) (Zheng et al., 2019).

### Statistical analysis

Basic statistics and Pearson’s correlation analysis were performed on the phenotypic data from each environment. Analysis of variance (ANOVA) was done using the SPSS (V22.0) statistical package (IBM SPSS, Armonk, NY, United States). SAS V8.0 (SAS Institute, Cary, North Carolina, United States)^2^ was used to calculate the best linear unbiased predictions (BLUP) and broad-sense heritability (*H*^2^) (Smith et al., 1998; Qin et al., 2016).

### Genetic linkage map construction and quantitative trait loci mapping

DNA was extracted from each line in the DH population and the respective parents using the CTAB method (Vijayalakshmi et al., 2010). The DH population and parents were genotyped with the Infinium wheat SNP 90K iSelect assay (Illumina Inc., San Diego, CA, United States) developed by the International Wheat SNP Consortium (Wang et al., 2014). IciMapping v4.0^3^ was used to construct a high-density genetic linkage map (Li et al., 2021). SNP markers with no recombination were placed into a single bin using the “BIN” function in IciMapping V4.0. The final markers were chosen with a minimum percentage of missing data and sorted into different groups with LOD (logarithm of odds) thresholds ≥ 8 using the “Grouping” function in JoinMap 4.0 (Li et al., 2021). QTL were detected using WinQTLCart version 2.5^4^ and composite interval mapping. The minimal LOD score to accept the presence of a QTL was set at 2.5. Stable QTL were stably detected in at least three datasets including the BLUP dataset. Major QTL were those that met these criteria in at least three environments (including the BLUP dataset as an environment) with more than 10% of explained phenotypic variation in at least one environment. QTL that were either within 1 cM of one another or shared common flanking markers were considered identical. The naming of QTL followed the International Rules of Genetic Nomenclature.^5^

To determine the physical position of identified QTL regions, a BLAST search in WheatOmics2.1^6^ was performed to align the QTL-associated peak and flanking SNP marker sequences (Ma et al., 2021).

### Validation for the major quantitative trait loci identified

To develop KASP tags from the peak marker SNP sequence of the major QTL, two specific primers (F1/F2) and a universal primer (R) were designed for each SNP. An F1 tail that could bind to induce FAM fluorescence and an F2 tail that could bind to induce HEX fluorescence were added to the specific sequences. KASP primers were designed by Polymarker^7^ and synthesized by Beijing Jiacheng Biotechnology Co., Ltd. The developed KASP markers were used to detect previously identified QTL in the DH118 × Jinmai 919 population as a means of validation. Following genotyping, the validation population was divided into two groups and differences in flag-leaf-related traits between the groups were assessed by *t*-tests in SAS V8.0 (Yang et al., 2022).

### Candidate gene analysis for the major quantitative trait loci identified

The genes within the target region were obtained using the genome browser (JBrowse) on the Triticeae Multi-omics website.^8^ Functional annotation and enrichment analysis of the genes were carried out in the GO (gene ontology) database using the R package cluster Profiler.

## Results

### A simple method to measure flag leaf thickness

For each of the five wheat cultivars, the thickness of each of the 20 segments was compared with the average of all segments. The leaf thickness of segments taken at two-thirds of the leaf length from tip to collar was 0.207–0.233 mm, which was close to the average thickness of the whole leaf (0.204–0.226 mm) (Supplementary Table 1). Thus, the leaf thickness from a single segment could be used as a measure of FLT (Table 1).

**TABLE 1:** Leaf thickness from a single segment compared with the average of 20 segments and calculated leaf volume compared to volume measured with the drainage method.

Variety	h (mm)			V (mm^3^)		
	Single segment	Average	δ (%)	Calculated	Drainage method	δ (%)
Liangxing 99	0.223 ± 0.011	0.220 ± 0.010	1.002	442.171 ± 11.426	466.667 ± 15.275	5.225
Jinmai 919	0.233 ± 0.011	0.226 ± 0.006	2.971	791.586 ± 7.975	816.667 ± 15.275	3.060
Jimai 22	0.222 ± 0.010	0.216 ± 0.016	2.895	533.733 ± 62.012	566.667 ± 70.237	5.752
Nongda 211	0.208 ± 0.008	0.204 ± 0.007	2.066	550.978 ± 35.578	596.667 ± 37.860	7.661
Taichang 29	0.207 ± 0.007	0.204 ± 0.002	2.621	535.697 ± 21.786	586.667 ± 30.551	8.592

δ (Relative Error) = ABS (Measured value-True value)/True value × 100%. The numbers in the table represent the values ± SD. Single segment represents 1 of 20 leaf segments, excised at approximately two thirds the distance from leaf tip to collar.

The new FLT assessment method was used to calculate FLV. The calculated FLV values were verified with the drainage method for assessing FLV. The average relative error was 3.060–8.592%, indicating that the method to calculate FLV in the present study was reliable (Supplementary Tables 2, 3). Also, the standard error for the calculated FLV was less than that of the drainage method, indicating the method was more precise.

### Phenotypic variation of flag-related traits

**FIGURE 1:**
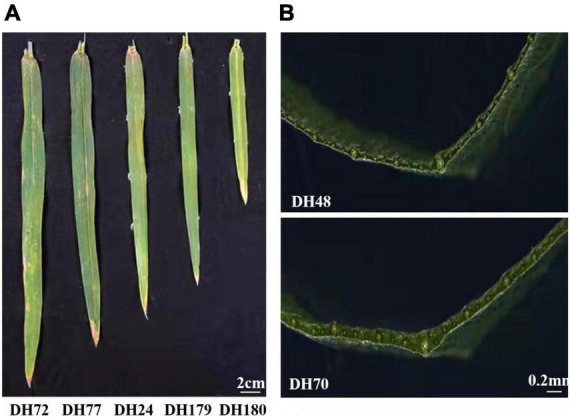
Phenotypes of selected DH lines. **(A)** The flag leaf size of lines from the Jinchun 7 × Jinmai 919 DH population. **(B)** Lines with different flag leaf thickness from the Jinchun 7 × Jinmai 919 DH population.

The phenotypic variation of the DH population and the parental lines was evaluated in five environments. Flag leaves of Jinmai 919 were larger than those of Jinchun 7 as measured by FLL, FLW, FLT, FLA, and FLV (Table 2) and the BLUP data. The flag-leaf-related traits of the DH population ranged from 10.50 to 29.78 cm for FLL, 1.06 to 2.83 cm for FLW, 0.10 to 0.29 mm for FLT, 10.60 to 59.32 cm^2^ for FLA, and 151.37 to 1311.56 mm^3^ for FLV (Figure 1). The *H*^2^ of five flag-leaf-related traits ranged from 0.69 to 0.86, indicating that these traits were significantly affected by genetic factors. The Pearson correlations among different environments were significant (Supplementary Table 4). All traits were normally distributed in the DH population (Supplementary Figure 1).

**TABLE 2:** Measurement of flag leaf-related traits in parents and the DH population.

Trait	Environment	Parents		DH population				
		Jinchun 7	Jinmai 919	Mean	SD	Min	Max	*H* ^2^
FLL (cm)	E1	23.24	23.73	21.93	2.15	16.63	28.23	0.86
	E2	17.78	19.23	20.91	2.48	15.78	29.78	
	E3	15.86	19.17	19.54	2.09	14.06	25.96	
	E4	13.35	15.55	15.12	2.04	10.50	25.90	
	E5	15.99	20.76	20.57	2.33	14.40	26.98	
	BLUP	18.90	21.42	18.86	1.46	15.83	24.70	
FLW (cm)	E1	2.07	2.14	2.05	0.24	1.47	2.83	0.86
	E2	1.94	1.94	2.04	0.22	1.42	2.68	
	E3	1.82	1.85	1.70	0.24	1.06	2.56	
	E4	1.53	1.54	1.55	0.16	1.12	2.07	
	E5	1.74	1.74	1.86	0.21	1.34	2.48	
	BLUP	1.90	2.05	1.77	0.14	1.42	2.28	
FLT (mm)	E1	0.18	0.19	0.19	0.03	0.10	0.29	0.69
	E2	0.20	0.21	0.19	0.03	0.10	0.29	
	E3	0.18	0.19	0.19	0.03	0.10	0.29	
	E4	0.17	0.16	0.17	0.03	0.11	0.24	
	E5	0.18	0.15	0.17	0.03	0.11	0.25	
	BLUP	0.18	0.21	0.18	0.01	0.15	0.22	
FLA (cm^2^)	E1	39.97	42.24	37.50	6.38	25.27	59.32	0.84
	E2	28.63	30.96	35.45	6.29	22.22	54.15	
	E3	23.96	29.49	27.63	5.03	14.04	49.21	
	E4	16.97	19.81	19.58	3.78	10.60	33.68	
	E5	23.10	29.98	31.96	5.86	18.41	52.49	
	BLUP	29.81	36.45	28.22	3.39	21.47	42.22	
FLV (mm^3^)	E1	719.38	802.61	703.00	178.33	347.87	1311.56	0.80
	E2	569.72	635.57	669.98	169.16	348.51	1252.92	
	E3	431.25	560.38	515.39	133.98	228.64	1088.10	
	E4	290.15	311.33	337.31	85.53	151.37	632.17	
	E5	414.70	438.12	549.95	129.09	284.17	986.56	
	BLUP	536.50	765.37	519.22	86.06	352.07	847.58	

SD, standard deviation; H^2^, broad-sense heritability; BLUP, best linear unbiased prediction.

### Correlation analysis for traits related to flag leaves and yield

**FIGURE 2:**
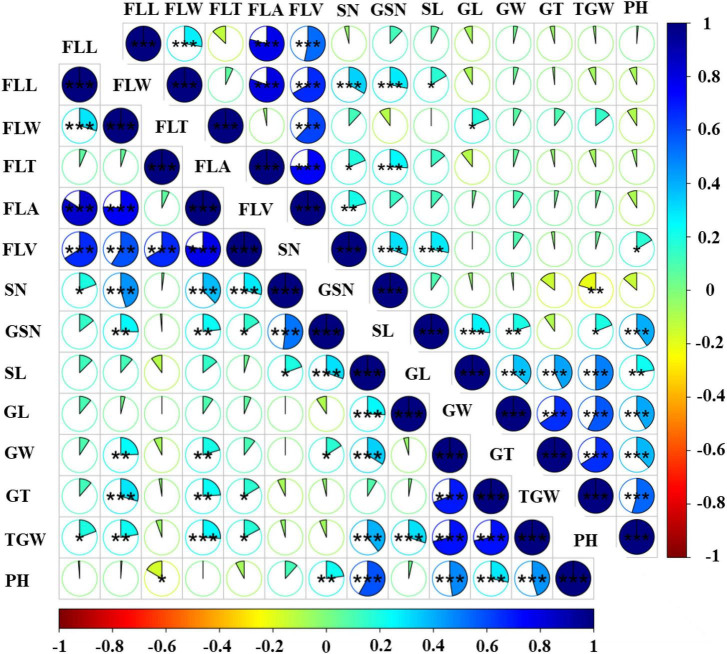
Phenotypic correlation coefficients between flag-leaf-related traits and yield related traits in the DH population grown under two water regimes. The lower left triangular matrix represents rainfed conditions, the upper right triangular matrix represents WW conditions, the sector area represents the correlation coefficients. *Significant at *P* < 0.05, **significant at *P* < 0.01, ***significant at *P* < 0.001.

The relationship between traits related to flag leaves and traits related to yield was analyzed under two different water regimes. For flag-leaf-related traits, FLL had a significant positive correlation with FLW (Figure 2 and Supplementary Table 5). Significant positive correlations were observed among FLL, FLW, and FLA. All four traits showed a significant positive correlation with FLV. However, FLL and FLW showed no correlations with FLT.

The relationship between flag-leaf-related traits and yield related traits was also evaluated. FLL showed highly significant positive correlations with SN and TGW in rainfed conditions, but FLL was negatively correlated with SN, GL, GT, TGW in WW conditions. Furthermore, FLW, FLA, and FLV had highly significant positive correlations with SN, GNS, GT, and TGW in both water regimes, and FLA and FLV showed significant positive correlations with GW in rainfed conditions. FLW, FLA, and FLV also had highly significant positive correlations with SN in both rainfed and WW conditions. However, different results were obtained for some pairs of traits under WW conditions. For example, in contrast to rainfed conditions, FLV and GNS were not correlated under WW conditions Also, although negative correlations were observed among FLT and GNS, SL, GW, GT, TGW, and PH in rainfed conditions, in WW conditions such correlations were only identified between FLT and GNS and PH.

### Linkage map construction

A high-density genetic linkage map for the DH population was constructed using marker data from the Wheat 90k SNP array. The total length of the map was 2,474 cM with an average genetic distance of 0.92 cM, including 2,700 SNP markers and covering all 21 chromosomes (Table 3). Respectively, the numbers of SNP markers in the A, B, and D genomes were 1,012, 1,128, and 560, the linkage lengths 789.59, 960.78, and 723.66 cM, and the average distances between markers 0.78, 0.85, and 1.29 cM. The D genome had the lowest marker coverage, the longest linkage group was 169.66 cM for chromosome 3B, and the shortest was 52.33 cM for chromosome 1A.

**TABLE 3:** Summary of linkage groups and marker statistics obtained from a 90K SNP array analysis of the Jinchun 7 × Jinmai 919 DH population.

Chromosome	No. of SNP markers	Length (cM)	Marker density (cM/marker)
1A	150	52.33	0.35
2A	182	120.54	0.66
3A	146	164.18	1.12
4A	120	121.27	1.01
5A	100	88.14	0.88
6A	131	125.02	0.95
7A	183	118.11	0.65
1B	183	140.92	0.77
2B	190	138.52	0.73
3B	153	169.66	1.11
4B	57	115.52	2.03
5B	194	158.16	0.82
6B	173	118.22	0.68
7B	178	119.78	0.67
1D	104	75.84	0.73
2D	96	72.08	0.75
3D	109	145.88	1.34
4D	35	90.55	2.59
5D	79	140.35	1.78
6D	34	80.56	2.37
7D	103	118.40	1.15
A genome	1,012	789.59	0.78
B genome	1,128	960.78	0.85
D genome	560	723.66	1.29
Total	2,700	2,474.03	0.92

### Quantitative trait loci mapping

Among all chromosomes except 4D and 5A, 79 QTL for flag-leaf-related traits were detected (Table 4 and Supplementary Table 6). These QTL explained 3.09–14.52% of the phenotypic variation in different environments. Favorable alleles of 33 QTL were derived from Jinchun 7 and favorable alleles of 46 QTL were derived from Jinmai 919.

**TABLE 4:** Stable quantitative trait loci (QTL) for flag-leaf-related traits detected in the DH population derived from Jinchun 7 × Jinmai 919.

Trait	QTL	Environment	Chr.	Peak marker	Genetic distance (cM)	Physical distance (Mb)	LOD	PVE (%)	Add
FLL	*QFLL-4A*	E1	4A	*BobWhite_c12128_187*	34.70–50.01	596.55–620.85	4.81	9.38	0.658
		E2	4A	*BS00041735_51*	28.92–42.11	593.48–607.48	4.95	9.15	0.757
		BLUP	4A	*BS00041735_51*	28.92–36.06	593.48–602.37	5.40	10.07	0.467
	*QFLL-7D*	E2	7D.1	*Excalibur_c12310_1611*	4.34–13.37	56.63–71.94	3.25	5.90	–0.602
		E5	7D.1	*Excalibur_c12310_1611*	4.34–14.67	56.63–73.25	5.85	12.08	–0.807
		BLUP	7D.1	*Excalibur_c12310_1611*	4.34–14.67	56.63–73.25	3.57	6.41	–0.368
FLW	*QFLW-1A*	E3	1A	*BS00110261_51*	29.74–31.09	440.54–475.93	3.52	6.68	–0.062
		E4	1A	*RAC875_c83508_72*	30.67–31.09	456.79–475.93	5.59	9.42	–0.050
		E5	1A	*BS00010130_51*	29.74–35.31	440.54–495.04	3.65	6.07	–0.053
		BLUP	1A	*RAC875_c83508_72*	30.67–31.09	456.79–475.93	2.50	3.75	–0.028
	*QFLW-4B*	E2	4B	*Kukri_c12661_326*	42.98–43.32	31.50–31.72	3.43	5.97	–0.054
		E5	4B	*CAP7_c10722_197*	35.13–43.32	24.96–31.72	2.76	5.92	–0.052
		BLUP	4B	*Kukri_c12661_326*	23.38–43.32	18.78–40.43	3.14	4.82	–0.031
	*QFLW-6A*	E2	6A	*RFL_Contig5166_1241*	68.79–71.11	69.34–102.10	4.05	7.01	0.059
		E3	6A	*BS00022992_51*	61.76–71.11	35.73–102.10	3.82	7.00	0.065
		E4	6A	*Excalibur_c19498_154*	61.76–66.47	35.73–74.42	7.74	14.52	0.062
		E5	6A	*RFL_Contig5166_1241*	68.57–71.11	69.34–102.10	3.14	5.00	0.048
		BLUP	6A	*BS00022992_51*	61.76–71.11	35.73–102.10	4.80	7.84	0.041
	*QFLW-7A.1*	E2	7A	*Tdurum_contig18052_599*	48.12–53.29	652.56–675.37	4.88	8.40	–0.064
		E4	7A	*Tdurum_contig18052_599*	49.62–52.68	659.55–674.24	2.57	4.31	–0.033
		E5	7A	*Tdurum_contig18052_599*	49.62–51.09	668.50–674.24	5.36	9.40	–0.065
		BLUP	7A	*Tdurum_contig18052_599*	50.00–52.68	659.55–674.24	4.95	8.11	–0.041
	*QFLW-7B.1*	E1	7B	*wsnp_CAP8_c3593_1773371*	58.49–69.18	108.32–593.01	3.90	6.98	0.064
		E3	7B	*RAC875_c60161_281*	58.49–69.88	108.32–598.33	3.65	6.65	0.063
		BLUP	7B	*RAC875_c60161_281*	58.88–70.16	108.32–687.62	4.32	7.01	0.038
	*QFLW-7D*	E1	7D.1	*D_GCE8AKX01EG53N_294*	33.29–37.63	129.75–256.41	4.83	10.15	–0.078
		E2	7D.1	*D_GDRF1KQ02H7HJS_109*	25.91–34.97	94.05–130.78	3.39	6.18	–0.057
		E3	7D.1	*RAC875_c62181_673*	35.87–46.11	149.71–174.23	3.86	7.09	–0.065
		BLUP	7D.1	*D_F5XZDLF02JMCIY_73*	27.59–46.27	94.05–190.19	3.74	7.03	–0.039
FLA	*QFLA-5D.1*	E1	5D.1	*CAP8_c6139_187*	31.60–38.81	342.69–368.11	3.93	7.31	1.739
		E3	5D.1	*CAP8_c6139_187*	27.68–48.41	305.18–381.73	3.11	6.12	1.286
		BLUP	5D.1	*CAP8_c6139_187*	31.60–48.41	342.69–381.73	3.10	5.60	0.804
	*QFLA-7A*	E2	7A	*Tdurum_contig33980_114*	53.63–55.76	636.22–678.88	2.71	4.92	–1.490
		E5	7A	*BobWhite_c5235_710*	53.63–54.35	648.28–678.88	4.01	6.90	–1.544
		BLUP	7A	*Tdurum_contig33980_114*	53.93–55.76	636.22–678.88	3.51	6.17	–0.869
	*QFLA-7D.1*	E1	7D.1	*Kukri_c40668_403*	23.35–35.72	85.28–132.10	4.92	10.72	–2.095
		E2	7D.1	*D_GDRF1KQ02H7HJS_109*	27.59–35.72	94.05–132.10	5.92	13.12	–2.302
		BLUP	7D.1	*D_GCE8AKX01EG53N_294*	27.59–35.73	94.05–132.10	4.70	9.49	–1.056
FLT	*QFLT-2B*	E2	2B	*Kukri_c64389_228*	42.18–52.36	38.34–66.37	2.79	7.18	–0.008
		E3	2B	*Kukri_c64389_228*	42.18–52.37	38.34–66.37	3.02	8.11	–0.009
		BLUP	2B	*Kukri_c64389_228*	42.18–52.38	38.34–66.37	2.82	7.59	–0.004
	*QFLT-6A*	E2	6A	*BobWhite_c3714_659*	22.23–27.96	6.62–12.49	3.86	8.09	–0.009
		E3	6A	*BobWhite_c3714_659*	22.23–27.96	6.62–12.49	3.48	7.31	–0.009
		E4	6A	*BobWhite_c3714_659*	22.52–23.95	8.02–9.28	3.30	6.77	–0.004
FLV	*QFLV-2A*	E1	2A	*BobWhite_c38516_148*	1.48–1.73	19.67–35.60	2.61	3.09	–31.798
		E2	2A	*GENE-1177_195*	0.00–0.95	18.34–36.45	4.29	7.90	–48.017
		BLUP	2A	*BobWhite_c38516_148*	1.37–1.73	19.67–35.60	3.27	6.63	–22.387
	*QFLV-7D*	E2	7D.1	*Kukri_c34950_1672*	42.55–58.49	311.74–538.29	5.85	11.50	–62.000
		E4	7D.1	*Kukri_c50621_436*	59.30–61.34	537.57–550.43	2.73	4.94	–19.455
		BLUP	7D.1	*Kukri_c34950_1672*	44.03–58.49	415.3–538.29	5.79	11.84	–31.719

PVE, phenotypic variance explained; LOD, logarithm of odds; Add, additive effect of a QTL with positive values indicating alleles from Jinchun 7 increased the trait scores, and negative values indicating alleles from Jinmai 919 increased the scores; BLUP, best linear unbiased prediction.

For FLL, 11 QTL were mapped to chromosomes 1B, 1D (2), 2B, 3B, 4A, 4B (2), 6B, 7B, and 7D, with individual QTL explaining 5.19–12.08% of the phenotypic variance. Among them, *QFLL-4A* and *QFLL-7D* were major QTL detected in three environments as well as with BLUP values, explaining 9.15–10.07% and 5.90–12.08% of the phenotypic variance, respectively. The additive effect values indicated that the positive alleles at *QFLL-4A* were from Jinchun 7 and those at *QFLL-7D* were from Jinmai 919. For the remaining 9 QTL, Jinchun 7 contributed five positive alleles and Jinmai 919 contributed four, and the range of phenotypic variation was 5.19–10.45%.

For FLW, 23 QTL were identified on chromosomes 1A, 2A, 2B (5), 2D (2), 3D, 4A (2), 4B, 5D, 6A, 7A (5), 7B (2), and 7D. These QTL explained 3.75–14.52% of the phenotypic variation in different environments. *QFLW-1A* and *QFLW-7A.1* were detected in four environments and their phenotypic variance ranged from 3.75 to 9.42% and 4.31 to 9.40%, respectively. *QFLW-4B* and *QFLW-7B.1* were detected in three environments and their phenotypic variance ranged from 4.82 to 5.97% and 6.65 to 7.01%, respectively. *QFLW-6A* was detected in five environments with phenotypic variance ranging from 5.00 to 14.52% and *QFLW-7D* was detected in four environments with phenotypic variance ranging from 6.18 to 10.15%. Among the 23 QTL, in 10 instances alleles that that increased FLW were from Junchun 7 and in 13 the positive alleles were contributed by Jinmai 919.

For FLA, 23 QTL were mapped to chromosomes 1D, 2A, 2B (3), 3A, 3B (2), 4A (2), 5D (2), 6A, 6D (2), 7A, 7B (2), and 7D (5), with individual QTL contributing 4.32–13.12% of the phenotypic variance. *QFLA-5D.1* and *QFLA-7A* were stably detected in three environments, explaining 5.60–7.31% and 4.92–6.90% of the phenotypic variance, respectively. *QFLA-7D.1*, a major QTL, was stably detected in three environments, explaining 9.49–13.12% of the phenotypic variance. For the 23 QTL, 11 of FLA-increase alleles were contributed by Jinchun 7 and 12 by Jinmai 919.

For FLT, six QTL were found on chromosomes 2A, 2B, 2D, 6A, 6B, and 7A, with the degree of phenotypic variation contribution ranging from 4.69 to 8.31%. The QTL *QFLT-2B* and *QFLT-6A* were observed in three environments and explained phenotypic variance from 7.18 to 8.11% and 6.77 to 8.09%, respectively. Additive effects indicated that, except for *QFLT-7A*, the QTL contributing to decreased FLA all came from Jinmai 919 alleles.

For FLV, 16 additive effect QTL were detected on chromosomes 1B, 1D, 2A, 2B, 2D, 4A, 5B, 5D, 6A (3), 7A (3), 7B, and 7D, and the phenotypic variance explained by individual QTL ranged from 3.09 to 11.84%. The QTL *QFLV-2A* was identified in three environments and explained phenotypic variance from 3.09 to 7.90%. *QFLV-7D* was a major QTL, which was stably detected in three environments and explained 4.94–11.84% of the phenotypic variance. The additive effect indicated that the allele contributing to the increase of FLV was from Jinmai 919. For the other 14 QTL, five of the FLV-increasing QTL were contributed by Jinchun 7.

**FIGURE 3:**
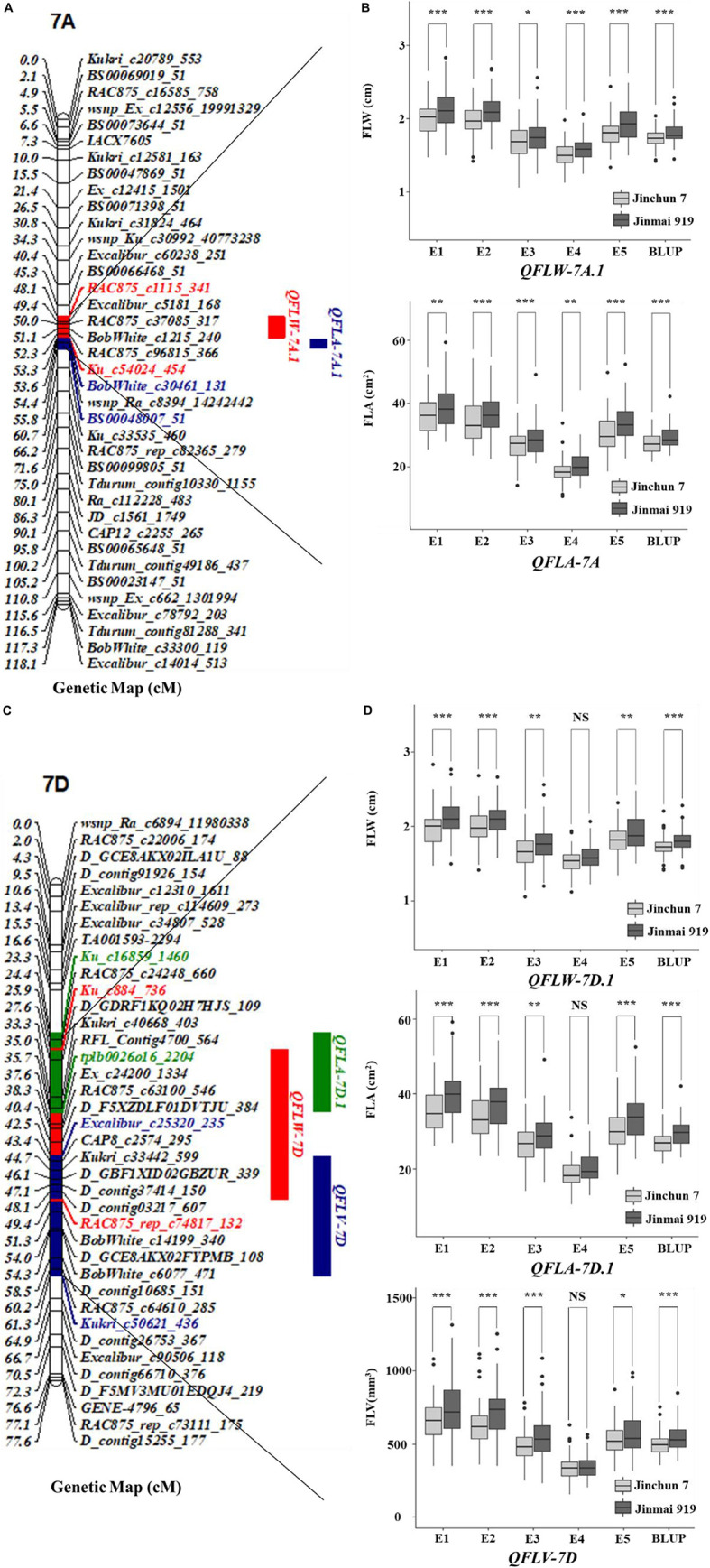
Genetic map of the major QTL *QFLW-7A.1*, *QFLA-7A.1*, *QFLW-7D*, *QFLA-7D.1*, *QFLV-7D*, and their effects. **(A,C)** Genetic map of 7A and 7D; **(B,D)** box plots of the effects of QTL on FLW, FLA, and FLV calculated after grouping the Jinchun 7 × Jinmai 919 DH population into two classes based on the allele of the flanking marker. *, **, and *** represent significance at *P* < 0.05, *P* < 0.01, and *P* < 0.001, respectively.

Remarkably, there were two co-localized regions for flag-leaf-related traits. *QFLW-7A.1* for FLW and *QFLA-7A.1* for FLA were co-located in the region of 636.22–678.88 Mb (Table 4 and Figure 3). Similarly, *QFLW-7D* for FLW, *QFLA-7D.1* for FLA, and *QFLV-7D* for FLV were co-located between markers *Ku_c16859_1460* and *Kukri_c50621_436*. To further validate these major QTL, we used the peak SNPs for the five QTL to evaluate their effects on the corresponding traits. The effect of *QFLW-7A.1* and *QFLA-7A.1* were significant (*P* < 0.05) in all six environments as well as BLUP values. The effect of *QFLW-7D*, *QFLA-7D.1*, and *QFLV-7D* were significant (*P* < 0.05) in all environments except for E4.

### Analyses of additive effects of the major quantitative trait loci

Generally, the higher the number of positive alleles, the larger the leaf. The linear relationship between phenotypic data and the number of positive alleles was analyzed.

**FIGURE 4:**
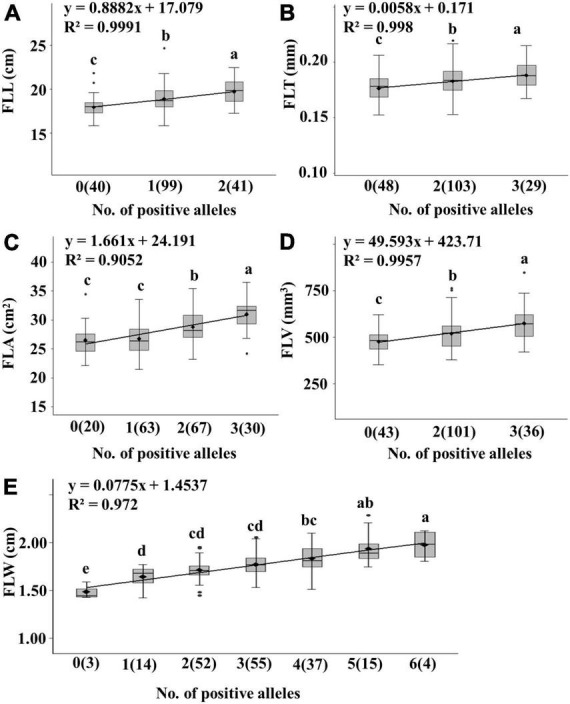
Linear regressions between number of positive alleles and trait values for five flag related traits in the Jinchun 7 × Jinmai 919 DH population **(A–E)**. The number of lines carrying the corresponding number of positive alleles are shown in brackets. The letter above the bars indicated comparisons result at the significant level 0.05.

We detected 15 stable QTL including two QTL for FLL (*QFLL-4A* and *QFLL-7D*), six QTL for FLW (*QFLW-1A*, *QFLW-4B*, *QFLW-6A*, *QFLW-7A.1*, *QFLW-7B.1*, and *QFLW-7D*), three QTL for FLA (*QFLA-5D.1*, *QFLA-7A*, and *QFLA-7D.1*), two QTL for FLT (*QFLT-2B* and *QFLT-6A*), and two QTL for FLV (*QFLV-2A* and *QFLV-7D*). The additive effects for each trait were further analyzed based on linked peak markers. The average traits value increased as the number of positive alleles increased (Figure 4 and Supplementary Table 7). DH lines with positive alleles at both QTL regions had an average FLL 1.78 cm (9.90%) greater than lines with contrasting alleles. Similarly, lines with more than two positive alleles showed significantly increased FLW. DH lines with positive alleles at all six QTL regions had an average FLW 0.49 cm (32.87%) greater than lines with contrasting alleles. DH lines with positive alleles at three QTL regions had an average FLA 0.52 cm^2^ (18.47%) greater than lines with contrasting alleles. DH lines with positive alleles at two QTL regions had an average FLT 0.01 mm (6.62%) thicker than lines with contrasting alleles. DH lines with positive alleles at two QTL regions had an average FLV 99.19 mm^3^ (20.87%) greater than lines with contrasting alleles.

### Co-location of for flag leaf thickness and flag leaf volume with yield related traits

**FIGURE 5:**
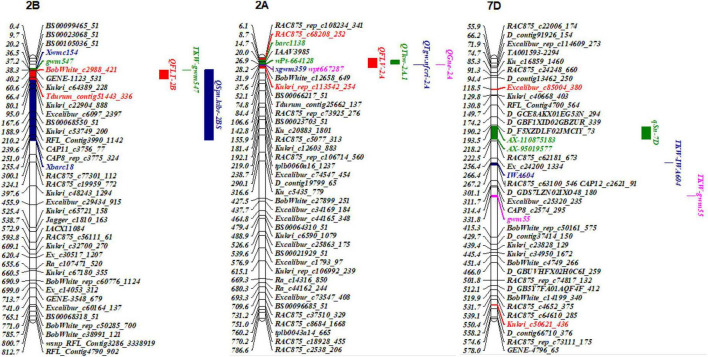
Physical map of the major QTL *QFLT-2B*, *QFLV-2A*, and *QFLV-7D*.

Hundreds of yield-related QTL have been mapped on all wheat chromosomes (Cao et al., 2020). *QFLT-2B* was mapped between *BobWhite_c2988_421* and *Tdurum_contig51443_336* and physically located between 38.34 and 66.37 Mb on chromosome 2B. It overlapped with loci *TKW-gwm547* for thousand kernel weight on 2B reported by Wang et al. (2012; Figure 5) *QFLT-2B* was near *QSpn.kibr-2BS* for SN that mapped between *Xwmc154* and *Xbarc18* and was physically located between 36.45 and 255.35 Mb on chromosome 2B, suggesting they may be allelic (Katkout et al., 2014). *QFLV-2A* mapped between *AC875_c68208_252* and *Kukri_rep_c113542_254* and was physically located between 8.71 and 37.61 Mb on chromosome 2A. It overlapped with *QTkw-2A.1* (Katkout et al., 2014) and *QTgw.nfcri-2A* (Wang et al., 2009) for TGW, and *QGne-2A* (Narjesi et al., 2015) for GNS, which were in the markers *barc1138*-*wPt664128*, *xgwm359*, and *wpt667287*-*Xgwm359*, respectively. In addition, *QFLV-7D* mapped between *BobWhite_rep_c50161_575* and *D_contig10685_151* and was physically located between 118.53 and 550.43 Mb on chromosome 7D, which indicated that this region may consist of several different QTL. In addition, *TKW-IWA604* (Zhang et al., 2018) and *TKW-gwm55* (Wang et al., 2012) were also located in the same interval. *qSn-7D* for SN mapped between *AX-110875183* and *AX-95019577* and was physically located between 193.47 and 218.18 Mb on chromosome 7D (Fan et al., 2019), which also overlapped with *QFLV-7D*.

### Quantitative trait loci validation

**FIGURE 6:**
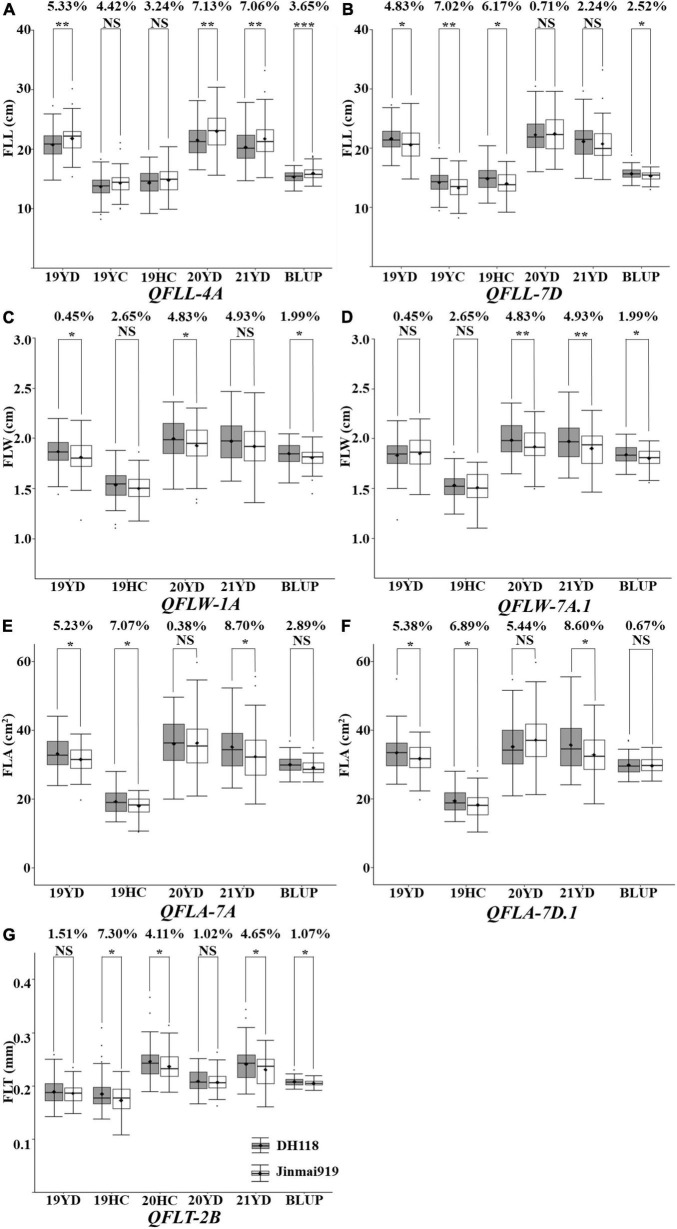
Validation of 11 stable quantitative trait loci (QTL) in the DH118 × Jinmai 919 population. Effects of *QFLL-4A* **(A)**, *QFLL-7D* **(B)** on flag leaf length and effects of *QFLW-1A* **(C)**, *QFLW-7A* **(D)** on flag leaf width and effects of *QFLT-2B* **(E)** on flag leaf thickness and effects of *QFLA-7A* **(F)** and *QFLA-7D* **(G)** on flag leaf area. Designations *, **, *** and NS represent *P* < 0.05, *P* < 0.01, *P* < 0.001 and no significant difference, respectively.

To further validate the fifteen major QTL, the KASP markers for each QTL were used to evaluate their effects on chlorophyll content in the DH118 × Jinmai 919 population. The KASP markers for *QFLW-4B*, *QFLW-6A*, *QFLW-7D*, and *QFLA-5D* were not polymorphic between DH118 and Jinmai 919, and thus the effects could not be evaluated. The remaining eleven QTL were evaluated. The effect of *QFLW-7B*, *QFLT-6A*, *QFLV-2A*, and *QFLV-7D* did not differ significantly between two groups in DH118 × Jinmai 919 population (Figure 6). The effect of other seven QTL, *QFLL-4A*, *QFLL-7D*, *QFLW-1A*, *QFLW-7A*, *QFLT-2B*, *QFLA-7A*, and *QFLA-7D*, were highly significant (*P* < 0.05) in more than three environments. According to marker profiles of *QFLL-7D*, *QFLW-1A*, *QFLW-7A*, *QFLT-2B*, *QFLA-7A*, and *QFLA-7D*, the lines with homozygous alleles from Jinmai919 had significantly higher (*P* < 0.05) values than those from Jinchun 7. The *QFLL-4A* lines homozygous for the Jinchun 7 alleles had significantly higher phenotypic values than those with the Jinmai919 alleles irrespective of QTL region, with differences in FLL ranging from 3.24 to 7.13%. The *QFLL-4A* lines homozygous for the Jinmai919 alleles had significantly higher phenotypic values than those with the Jinchun 7 alleles irrespective of QTL region, with differences in FLL ranging from 0.71 to 7.02%. Lines with the positive allele from *QFLW-1A* and *QFLW-7A* had significantly greater FLW ranging from 2.29 to 3.52% and 0.45 to 4.93%, respectively. The FLT of lines with the *QFLT-2B* allele was significantly higher than for lines lacking the *QFLT-2B* allele, and the difference was between 1.07 and 7.30%. Lines with the positive allele for *QFLA-7A* and *QFLA-7D* had significantly greater FLA.

### Marker development

To apply important QTL associated with flag-leaf-related traits to wheat breeding and gene cloning, seven SNPs were identified, *wsnp_Ex_c19773_28772235* (4A) linked to *QFLL-4A* and *D_contig26694_169* (7D) linked to *QFLL-7D* for FLL, *wsnp_Ex_c572_1138770* (1A) linked to *QFLW-1A* and *wsnp_BQ160404A_Ta_1_1* (7A) linked to *QFLW-7A.1* for FLW, *wsnp_Ra_c265_560747* (2B) linked to *QFLT-2B* for FLT, *wsnp_BQ160404A_Ta_1_1* (7A) linked to *QFLA-7A* and *IAAV2530* (7D) linked to *QFLA-7D.1* for FLA (Supplementary Table 8).

### Putative candidate genes

A total of 6,007 genes were identified within the seven major QTL, 1,059 genes in *QFLL-4A*, 503 genes in *QFLL-7D*, 1,036 genes in *QFLW-1A*, 516 genes in *QFLW-7A*, 767 genes in *QFLT-2B*, 1,071 genes in *QFLA-7A*, and 1,055 genes in *QFLA-7D* (Supplementary Table 9). Among these genes in each interval, some were related to leaf growth and development. A cluster of 226 genes harboring an F-box domain may be of particular interest because they were reported to participate in hormone signal transduction, which affects leaf development (Song et al., 2012). *TraesCS4A03G0838400LC* and *TraesCS7D03G0218200* encode purple acid phosphatase, which are involved in leaf senescence in *Arabidopsis* (Robinson et al., 2012; Tu et al., 2021). Seventeen ethylene-responsive transcription factors were reported to regulate leaf development (Koyama, 2014).

## Discussion

### A Simple and accurate method to measure flag leaf thickness

Previous studies generally used the drainage method to measure leaf volume. However, since wheat leaf volume is relatively small and difficult to measure the results using this method are variable. Leaf thickness is the main parameter of flag leaf volume. At present, leaf thickness is mainly measured through a plant leaf parameter instrument with the help of modern sensing technology and a direct contact method. Leaf thickness can change during extrusion measurement, which introduces errors. Wheat flag leaves gradually thins from base to tip, further complicating the measurement of wheat flag leaf volume. In the present study, leaf volume was measured by incorporating the use of a Leica microscope and the image processing software Image J to measure leaf thickness. Leaf thickness at two-thirds of the leaf length from tip to collar was found to represent the average leaf thickness, and this discovery enabled a more accurate calculation of leaf volume. Leaf volume as measured with the more laborious drainage method was consistent with results from the new method. This new high-throughput method for measuring leaf volume will have value in future research on leaf volume in cereal crops.

### Nine novel stable quantitative trait loci for flag-leaf-related traits were detected

Fifteen major QTL for flag-leaf-related traits were detected in the present study. To compare the intervals of the QTL detected with those identified previously, these QTL were physically mapped on target chromosomes in Chinese Spring. *QFLL-7D* mapped between *D_GCE8AKX02ILA1U_88* and *RAC875_c44223_285* and was physically located between 56.63 and 73.25 Mb on chromosome 7D. It overlapped with *QFLL-7D.2*, which mapped between 72.95 and 93.50 Mb (Hu et al., 2020). *QFLW-1A* mapped between *Excalibur_c9662_70* and *Tdurum_contig44112_1609* and was physically located between 102.14 and 556.49 Mb on chromosome 1A. It overlapped with *QFLW-1A*, which mapped to 511.45 Mb (Yan et al., 2020). *QFLW-6A.1* overlapped with *QFlw.sicau-6A.2* as reported by Ma et al. (2020) and was close to *QFLW-6A* (Yan et al., 2020). *QFLW-7A.1* overlapped with a genetic region reported by Yang et al. (2016). *QFLW-7B.1* was located near *QFlw.sicau-7B* (Ma et al., 2020). *QFLW-7D.1* was located near QTL *QFlw.cau-7D*, which was detected in all four environments tested (Wu et al., 2016). Comparison of physical intervals suggested that no previously reported QTL overlapped with *QFLL-4A*, *QFLW-4B*, *QFLA-5D.1*, *QFLA-7A*, and *QFLA-7D.1.* Thus, these five QTL are likely novel loci. There are few reports of QTL for FLT and FLV in wheat, so *QFLT-2B*, *QFLT-6A*, *QFLV-2A*, and *QFLV-7D* are also likely novel.

### Leaf thickness is of great significance to gain yield

Leaf thickness is an important leaf trait as it affects light energy utilization and yield. Two QTL for FLT, *QFLT-2B* and *QFLT-6A*, were detected in the Jinchun 7 × Jinmai 919 DH population. One of the QTL for FLT was co-located with QTL for yield related traits. *QFLT-2B* overlapped with the previously identified loci *TKW-gwm547* for TKW and *QSpn.kibr-2BS* for SN, indicating that these loci have either a pleiotropic effect or are closely linked (Wang et al., 2012; Katkout et al., 2014). Breeding for optimum flag leaf size is thought to be a good approach to improve grain yield by reducing shading and disease conduciveness within the plant population. However, there is an upper limit to increasing leaf area for increased grain yield. Therefore, increasing leaf thickness through breeding offers another approach to increase the photosynthetic potential and grain yield. In addition, the SNPs for FLT identified in the present study could be converted to KASP markers and used in molecular marker-assisted breeding in the future.

## Data availability statement

The original contributions presented in this study are included in the article/Supplementary material, further inquiries can be directed to the corresponding authors.

## Author contributions

YW, LQ, CY, XL, JJZ, and BW: data curation. LQ, XZ, PL, and JZ: methodology. YW and LQ: writing—original draft. XZ, PL, and JZ: writing—review and editing. All authors contributed to the article and approved the submitted version.

## Footnotes

1
https://imagej.nih.gov/ij/

2
https://www.sas.com

3
https://isbreedingen.caas.cn/software/qtllcimapping/294605.htm

4
https://brcwebportal.cos.ncsu.edu/qtlcart/WQTLCart.htm

5
https://wheat.pw.usda.gov/ggpages/wgc/98/Intro.htm

6
http://wheatomics.sdau.edu.cn/blast/blast.html

7
http://www.polymarker.info/

8
http://wheatomics.sdau.edu.cn
